# The urokinase receptor-derived cyclic peptide [SRSRY] suppresses neovascularization and intravasation of osteosarcoma and chondrosarcoma cells

**DOI:** 10.18632/oncotarget.9976

**Published:** 2016-06-13

**Authors:** Vincenzo Ingangi, Katia Bifulco, Ali Munaim Yousif, Concetta Ragone, Maria Letizia Motti, Domenica Rea, Michele Minopoli, Giovanni Botti, Giuseppe Scognamiglio, Flavio Fazioli, Michele Gallo, Annarosaria De Chiara, Claudio Arra, Paolo Grieco, Maria Vincenza Carriero

**Affiliations:** ^1^ Neoplastic Progression Unit, Department of Experimental Oncology, IRCCS Istituto Nazionale Tumori “Fondazione G. Pascale”, Naples, Italy; ^2^ SUN Second University of Naples, Naples, Italy; ^3^ Department of Pharmacy, University Federico II, Naples, Italy; ^4^ University ‘Parthenope’, Naples, Italy; ^5^ Animal Facility, IRCCS Istituto Nazionale Tumori “Fondazione G. Pascale”, Naples, Italy; ^6^ Pathology Unit, IRCCS Istituto Nazionale Tumori “Fondazione G. Pascale”, Naples, Italy; ^7^ Surgery Unit, IRCCS Istituto Nazionale Tumori “Fondazione G. Pascale”, Naples, Italy

**Keywords:** urokinase receptor, formyl peptide receptor type 1, osteosarcoma, chondrosarcoma, peptides

## Abstract

The receptor for the urokinase-type plasminogen activator (uPAR) is a widely recognized master regulator of cell migration and uPAR_88–92_ is the minimal sequence required to induce cell motility and angiogenesis by interacting with the formyl peptide receptor type 1 (FPR1). In this study, we present evidence that the cyclization of the uPAR_88–92_ sequence generates a new potent inhibitor of migration, and extracellular matrix invasion of human osteosarcoma and chondrosarcoma cells expressing comparable levels of FPR1 on cell surface. *In vitro*, the cyclized peptide [SRSRY] prevents formation of capillary-like tubes by endothelial cells co-cultured with chondrosarcoma cells and trans-endothelial migration of osteosarcoma and chondrosarcoma cells. When chondrosarcoma cells were subcutaneously injected in nude mice, tumor size, intra-tumoral microvessel density and circulating tumor cells in blood samples collected before the sacrifice, were significantly reduced in animals treated daily with i.p-administration of 6 mg/Kg [SRSRY] as compared to animals treated with vehicle only. Our findings indicate that [SRSRY] prevents three key events occurring during the metastatic process of osteosarcoma and chondrosarcoma cells: the extracellular matrix invasion, the formation of a capillary network and the entry into bloodstream.

## INTRODUCTION

The development of metastases is a multistep process that requires active and specifically localized extracellular proteolysis as well as the activation of a series of physiological and biochemical processes that govern the migration from the primary tumor site, the invasion through the basement membrane, the entry of metastatic cells into the blood vessels and finally localization to the second site. Despite significant progress regarding potential therapeutic targets aimed at improving survival, patients affected by osteosarcoma or chondrosarcoma frequently die for systemic spread of the disease, mainly to the lungs [1–2]. Thus, elucidating the mechanisms controlling metastasis is important for improving outcome of patient with osteosarcoma or chondrosarcoma. Both diseases are characterized by high neovascularization and a high propensity to metastasize through bloodstream [3], but the cellular processes that lead to their interactions with endothelium and subsequent invasion through endothelial environment are poorly understood.

The urokinase receptor (uPAR) is emerging as a cell surface-associated molecule relevant to cancer invasion and metastasis [4–5]. The clinical relevance of uPAR as a prognostic marker, when measured in tumor tissues and/or plasma, has been demonstrated in various cancer diseases, including sarcomas and chondrosarcomas [6–8]. Interestingly, it has been documented in a mouse model of osteosarcoma that silencing expression of uPAR results in a significant reduction of metastasis to lung [9].

The uPAR consists of three domains (D1, D2, and D3), anchored to the cell surface through a carboxy-terminal glycosyl-phosphatidyl-inositol anchor [10]. Full uPAR or fragments thereof (deriving from cleavages at protease-sensitive regions of the receptor) on cell surface may be released in soluble forms in plasma and/or urine. When expressed on cell surface, uPAR promotes cell-associated proteolysis by binding to uPA, which locally converts plasminogen into active plasmin, thus favoring tissue invasion and metastasis [4–5]. Plasmin generated by uPA or uPA itself can cleave intact uPAR (D1D2D3), releasing D1. The remaining GPI-anchored D2D3 can be left on cell surface or be secreted in the extracellular milieu following cleavage of the anchor [11]. Some years ago, we found that chondrosarcoma cells produce and release in the culture medium soluble forms of uPAR, including the intact D1D2D3 and the D2D3 fragment [8]. Ligand-engaged uPAR also acts as a potent regulator of tumor cell migration and matrix attachment, independently of its catalytic activity [4–5]. We and others have shown that signaling occurs through the assembly of uPAR in composite regulatory units with extracellular matrix (ECM) proteins such as vitronectin, and with transmembrane receptors like the G protein-coupled formyl-peptide receptors (FPRs) as well as integrins [12–20].

A crucial signaling region is the protease sensitive region linking D1 and D2 domains (uPAR_84–95_) which retains chemotactic activity [15, 16]. Its minimal active ^88^Ser-Arg-Ser-Arg-Tyr^92^ sequence is able to trigger cell migration and angiogenesis *in vitro* and *in vivo,* even in the form of synthetic linear Ser-Arg-Ser-Arg-Tyr peptide (SRSRY) [17, 21]. Mechanistically, uPAR_88–92_ sequence promotes cell motility by interacting with FPR1 which, in turn, triggers vitronectin receptor activation with an inside-outside type of mechanism [17]. The X-ray studies have shown that the three uPAR domains pack together into a concave structure that binds uPA, and that the domain boundary between uPAR D1-D2 is more flexible than the D2-D3 domain boundary [22–24]. Thus, we reasoned that cyclization of the Ser-Arg-Ser-Arg-Tyr peptide could reduce conformational flexibility of its linear form, thus generating a new, more stable peptide that could regulate uPAR_88–92_-dependent functions. We found that both linear SRSRY and cyclized [SRSRY] peptides compete with fMLF for binding to FPR type 1 (FPR1). However, these peptides exert opposite effect on monocyte motility, the linear SRSRY promotes cell migration, while the peptide [SRSRY] inhibits cell migration in a dose-dependent manner, with IC_50_ value of 0.01 nM. Unlike the linear peptide SRSRY, [SRSRY] displays a long-time resistance to enzymatic digestion in serum and prevents trans-endothelial migration of monocytes [25]. *In vivo*, [SRSRY] reduces intestinal inflammation diminishing recruitment of inflammatory monocytes to the inflamed tissue [26].

In the present study we explored the possibility that [SRSRY] may affect trans-endothelial migration of osteosarcoma and chondrosarcoma cells. Herein, we show that the cyclization of the uPAR_88–92_ sequence generates a potent inhibitor of migration, and extracellular matrix invasion of human osteosarcoma and chondrosarcoma cells expressing comparable levels of FPR1 on cell surface. Interestingly, [SRSRY] inhibits tube formation of endothelial cells co-cultured with chondrosarcoma cells and trans-endothelial migration of osteosarcoma and chondrosarcoma cells. Furthermore, [SRSRY] exerts anti-metastatic effect reducing *in vivo* vascular infiltration by chondrosarcoma cells.

## RESULTS

### The peptide [SRSRY] inhibits migration and invasion of osteosarcoma and chondrosarcoma cells expressing comparable levels of FPR1

**Figure 1:**
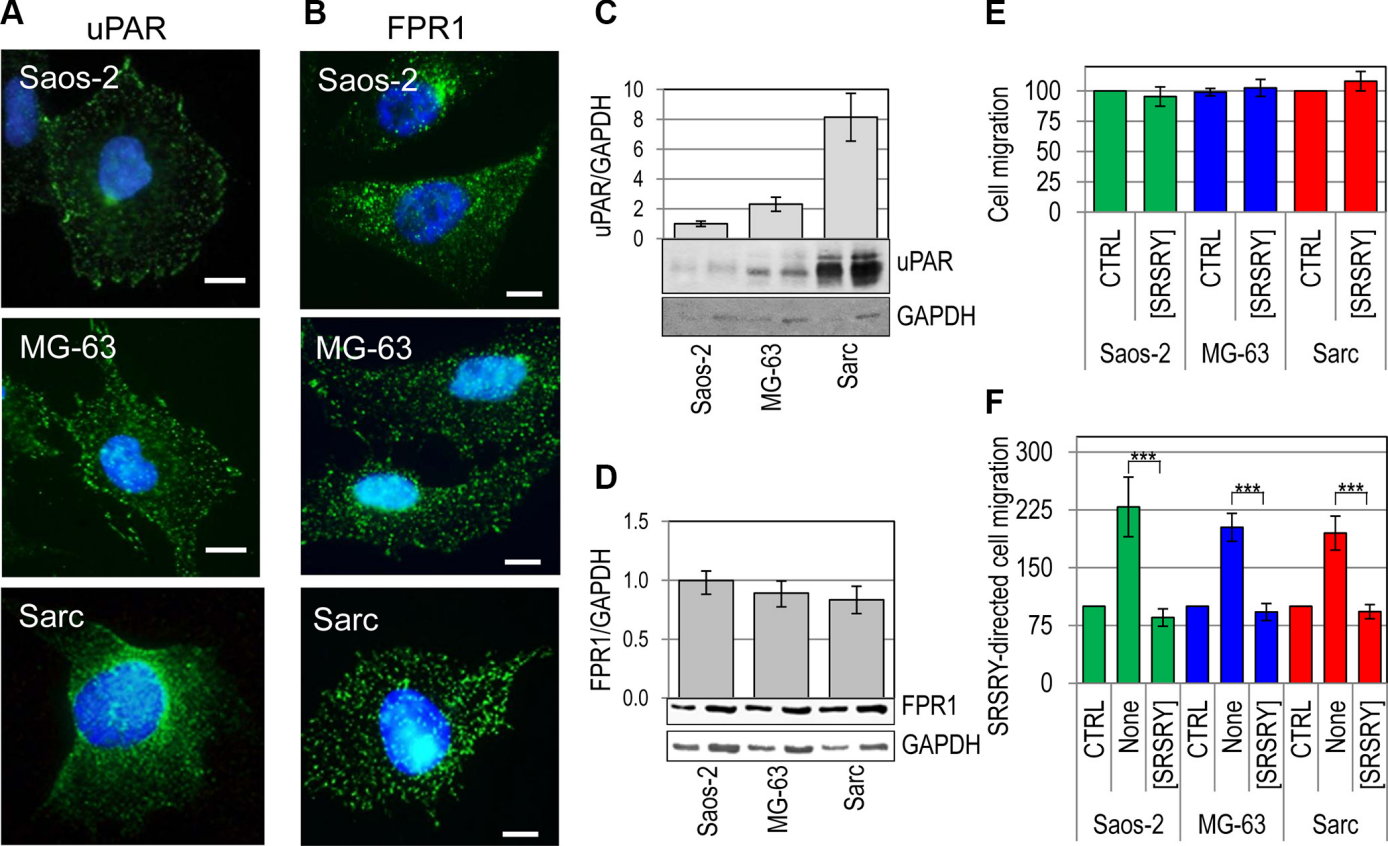
Inhibitory effect of [SRSRY] on migration of FPR1expressing osteosarcoma and chondrosarcoma cells (**A–B**) Representative images of human osteosarcoma Saos-2 and MG-63 cells, and human chondrosarcoma Sarc cells incubated with 2 μg/mL R4 anti-uPAR monoclonal antibody (A) or 1:100 anti-FPR1 polyclonal antibody 2 h at 23°C, exposed to Alexa 488-coniugated F(ab')2 fragment of rabbit anti-mouse IgG or goat anti-rabbit IgG for 40 min at 23°C and visualized by a fluorescence inverted microscope. Nuclei were stained blue with DAPI. Scale bar: 10 μm. Original magnification: 1000 x. (**C–D**) Whole cell lysates (20 and 40 μg/sample) from Saos-2, MG-63 and Sarc cells were resolved on a 10% SDS-PAGE under unreducing (C) or reducing conditions (D), followed by Western blotting with 1 μg/mL R4 anti-uPAR monoclonal antibody (C) or 1 μg/mL anti-FPR1 polyclonal antibody (D) and 0.2 μg/mL anti-GAPDH polyclonal antibody as loading control. The enclosed bar graphs show the average quantification of the uPAR/GAPDH (C) and FPR1/GAPDH (D) content from 3 independent experiments. (**E–F**) Saos-2, MG-63 and Sarc cells were allowed to migrate for 4 h at 37°C in 5% CO_2_ in Boyden chambers toward DMEM (CTRL), or 10 nM [SRSRY] (E), DMEM (CTRL) or 10 nM SRSRY, in the absence (None) or the presence of 10 nM [SRSRY] (F). In all cases, the extent of cell migration was expressed as a percentage of the basal cell migration assessed toward serum-free medium, considered as 100% (CTRL). Data are expressed as the mean ± SD of three independent experiments, performed in triplicate. ***Statistical significance calculated against the positive control (None) with *p < 0.0001*.

**Figure 2:**
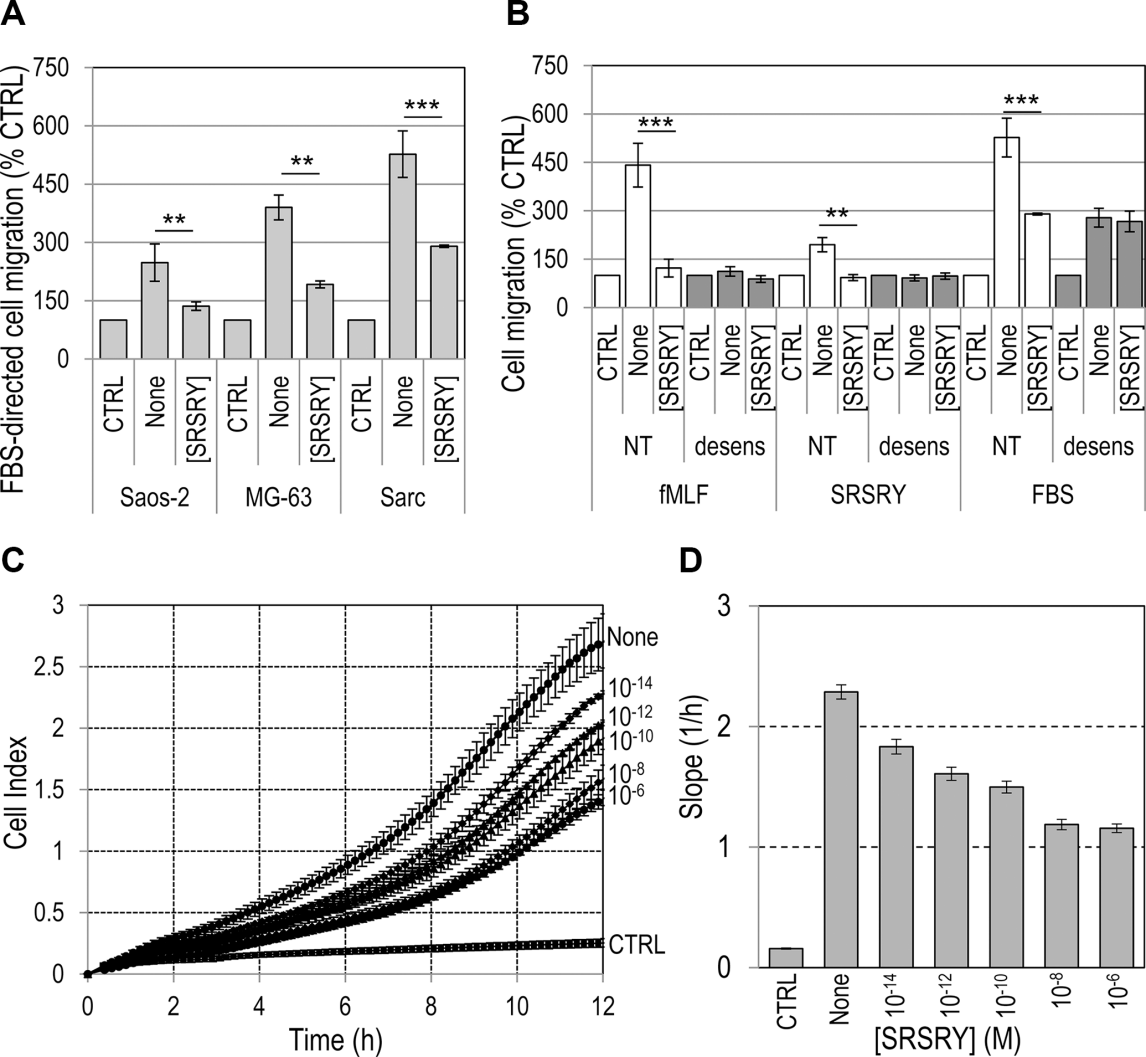
Dose-dependent inhibitory effect of [SRSRY] on migration of osteosarcoma and chondrosarcoma cells (**A**) The indicated cell lines were allowed to migrate toward 10% FBS (None) plus/minus 10 nM [SRSRY] in Boyden chambers for 4 h. (**B**) Sarc cells were exposed to diluents (NT) or desensitized with 100 nM fMLF for 30 min at 37°C and then allowed to migrate toward 10 nM fMLF, 10 nM SRSRY or 10% FBS without (None) or with 10 nM [SRSRY] in Boyden chambers for 4 h. In all cases, for quantitative analysis of cell migration, the basal value assessed in the absence of chemoattractants (CTRL) was taken as 100% and all values were reported relative to that. Data are the means ± SD of three independent experiments, performed in triplicate. Statistical significance calculated against None with **p < 0.001; ***p < 0.0001; (**C**) Migration of Sarc cells monitored in real-time for 12 h as changes in cell index by the xCELLigence system. Cells were seeded in CIM-16-well plates and allowed to migrate at 37°C, 5% CO_2_, toward 10% FBS (None) or 10% FBS plus increasing concentration of [SRSRY]. (**D**) Slopes represent the change rate of cell index generated in a 1–12 h time frame. Data represent mean ± SD from a quadruplicate experiment.

We have recently found that the cyclized peptide SRSRY ([SRSRY]) inhibits in a dose-dependent manner directional migration of rat basophilic leukemia RBL-2H3/ETFR cells expressing high levels of constitutively activated FPR1. [SRSRY] exerts inhibitory effect by preventing uPAR/FPR1 interaction and, consequently, agonist-triggered FPR1 activation [25]. To investigate whether [SRSRY] affects the motility of osteosarcoma and chondrosarcoma cells, cell migration assays were carried out in Boyden chambers using two human osteosarcoma Saos-2 and MG-63 cell lines and a human chondrosarcoma Sarc cell line derived from a primary culture [8]. Saos- 2, MG-63 and Sarc cells express low, medium and high levels of uPAR, respectively, and comparable levels of FPR1 as shown by immunofluorescence (Figure 1A–1B) and Western blot analysis (Figure 1C–1D). The peptide [SRSRY] failed to trigger migration of all tested cell lines when used as chemoattractant at 10 nM concentration in Boyden chambers (Figure 1E). However, when the uPAR derived linear peptide SRSRY was employed to produce the chemotactic gradient, all cell lines were able to respond to mitogen stimulus, and the addition of equimolar concentration of [SRSRY] (10 nM) reduced to the basal level their motility (Figure 1F). These data well agree with the notion that the linear peptide SRSRY promotes cell motility by interacting with FPR1 whereas its cyclic form inhibits cell migration by preventing SRSRY- or fMLF-triggered FPR1 activation [17, 25]. They also highlight the involvement of FPR1 in the migration ability of osteosarcoma and chondrosarcoma cells. To evaluate the effect of [SRSRY] in a system more representative of the *in vivo* context, cells were tested for their ability to migrate toward serum which is a source of many chemoattractants. Not surprisingly, 10% FBS elicited a considerable cell migration of Saos-2, MG-63 and Sarc cells reaching 248%, 390% and 527% of the basal cell migration, respectively. The addition of 10 nM [SRSRY] to the lower compartment of Boyden chambers, reduced cell migration of Saos-2, MG-63 and Sarc cells by 45%, 58% and 55%, respectively. These data again agree with the comparable expression levels of FPR1 on Saos-2, MG-63 and Sarc cells since, despite their different ability to migrate toward serum, [SRSRY] reduced by about 50% their cell motility (Figure 2A). To further confirm the requirement of FPR1 in the [SRSRY] inhibitory effect, a subset of cell migration experiments were performed using Sarc cells desensitized with 100 nM fMLF as described [21]. As expected, desensitized cells failed to move towards 10 nM SRSRY or 10 nM fMLF, and retained the ability to respond to serum containing chemoattractants, although to a minor extent as compared to untreated cells (Figure 2B). In all cases, [SRSRY] did not exert inhibitory effect on basal as well as on FBS-dependent migration of desensitized cells (Figure 2B) and reduced cell migration toward SRSRY or 10 nM fMLF to the basal level. All together, these findings indicate that [SRSRY] inhibits only FPR1-mediated cell motility.

**Figure 3:**
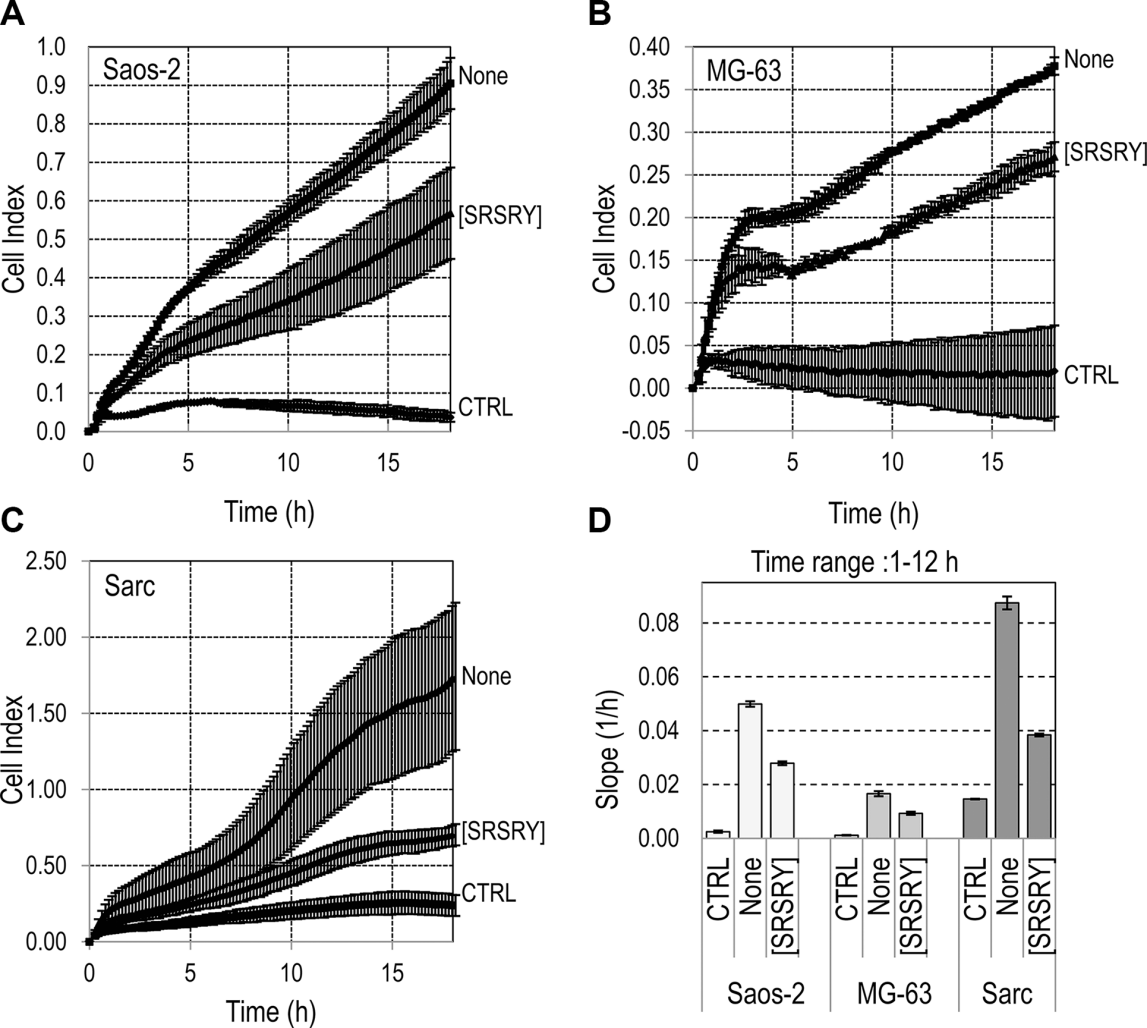
Effect of [SRSRY] on matrigel invasion by osteosarcoma and chondrosarcoma cells (**A–C**) The indicated cells re-suspended in serum-free medium (CTRL) or growth medium without (None) or with 10 nM [SRSRY] were seeded on polymerized matrigel in E-16-well plates and allowed to invade matrigel for 18 h. Microelectrodes detect impedance changes which are proportional to the number of cells that cross matrigel and are expressed as cell index. Data represent mean ± SD from quadruplicate experiments. (**D**) Slopes represent the change rate of cell index generated in a 1–12 h time frame.

When cell migration of Sarc cells was monitored in real time using the xCELLigence RTCA technology, Sarc cells showed a great ability to migrate toward serum in agreement with results obtained in Boyden chambers. The addition of [SRSRY] reduced their migration in a dose-dependent manner (Figure 2C). Slopes representing the change rate of cell index generated in the time ranges relative to exponential phase curves, revealed that inhibition starts in the fM range, it seems to level off in the nM range and reaches an overall 50% reduction at ~100 pM (Figure 2D). Cell migration is a prerequisite for cancer invasion. Therefore, we investigated whether [SRSRY] prevents matrigel invasion of osteosarcoma and chondrosarcoma cells using the xCELLigence RTCA technology. Saos-2, MG-63 or Sarc cells re-suspended in serum free (CTRL) or growth medium with or without 10 nM [SRSRY] were seeded on polymerized matrigel. Matrigel invasion was monitored in real-time for 18 h as cell index changes due to the adhesion of invading cells to microelectrodes. As shown in Figure 3, all cell lines were able to cross matrigel, although to a different extent. [SRSRY] reduced matrigel invasion of Saos-2 (A), MG- 63 (B) and Sarc (C) cells, by about 44%, 43% and 56%, respectively (Figure 3D).

### The peptide [SRSRY] prevents endothelial tube formation in a co-culture assay

**Figure 4:**
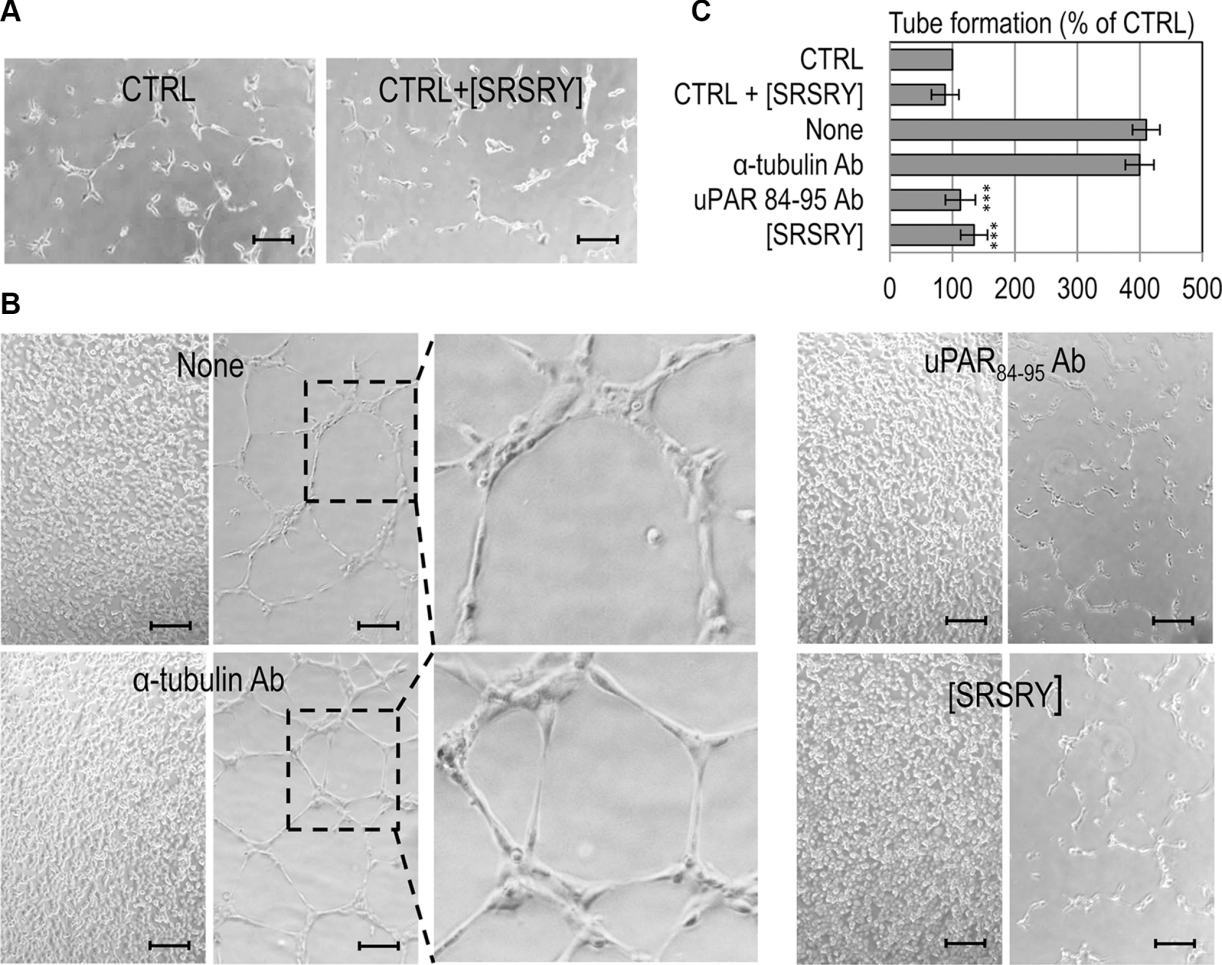
Inhibitory effect of [SRSRY] on tube formation of endothelial cells co-cultured with Sarc cells Sarc cells were grown to confluence and allowed to release pro-angiogenic factors in serum free medium for 18 h. Then, HUVEC plated on matrigel in an intercup chamber were layered onto EBM empty well (CTRL) or Sarc cells in the presence or in the absence of 10 nM [SRSRY], 4 μg/mL anti-uPAR_84-95_ Ab or 4 μg/mL anti-α-tubulin control Ab. (**A–B**) Representative pictures were taken with an inverted microscope. Scale bar: 100 μm. Original magnifications: 50×. Dashed boxes enclose areas shown at higher magnification. (**C**) Quantitative analysis of tube formation was calculated as a percentage of tubes formed by cord-like structures exceeding 100 μm in length, counted in the absence of any angiogenic stimulus and considered as 100% (CTRL). Data represent means ± SD of three independent experiments performed in duplicate. Statistical significance was calculated against None with ***p < 0.0001.

We have previously documented that: ì) the short Ser^88^-Arg-Ser-Arg-Tyr^92^ chemotactic sequence of SuPAR stimulates *in vitro* and *in vivo* angiogenesis even in the form of synthetic linear peptide SRSRY [21]; ìì) chondrosarcoma Sarc cells release a large amount of SuPAR in the medium [8]; ììì) peptide inhibitors of the uPAR/FPR1 interaction inhibit angiogenesis [27]. Therefore, we investigated whether Sarc cells promote endothelial tube formation in a non-contact co-culture system and whether [SRSRY] exerts some effect. Sarc cells were grown to confluence and allowed to release pro-angiogenic factors in serum free medium for 18 h. Then, HUVECs plated on matrigel in an intercup chamber were put onto Sarc cells and exposed to their secretion products for 4 h. Quantitative analysis of tube formation was expressed as a percentage of tubes formed by cord-like structures exceeding 100 μm in length, counted in the presence of serum free medium, considered as 100% (CTRL). In the absence of Sarc monolayer, endothelial cells failed to form cord-like structures either in the absence (CTRL) or in the presence of 10 nM [SRSRY] (CTRL+[SRSRY]), (Figure 4A, 4C). *Vice-versa*, Sarc cells stimulated the formation of tube-like structures (None) which were reduced almost to basal levels by blocking anti-uPAR_84–95_ polyclonal antibodies [21] but not anti α-tubulin antibodies (Figure 4B–4C), indicating that the Sarc-triggered proangiogenic effect is mostly due to release in the conditioned medium of SuPAR. As a result, the addition of 10 nM [SRSRY] reduced endothelial capillary-like structures by 67% (Figure 4B–4C).

### The peptide [SRSRY] reduces trans-endothelial migration of osteosarcoma and chondrosarcoma cells

**Figure 5:**
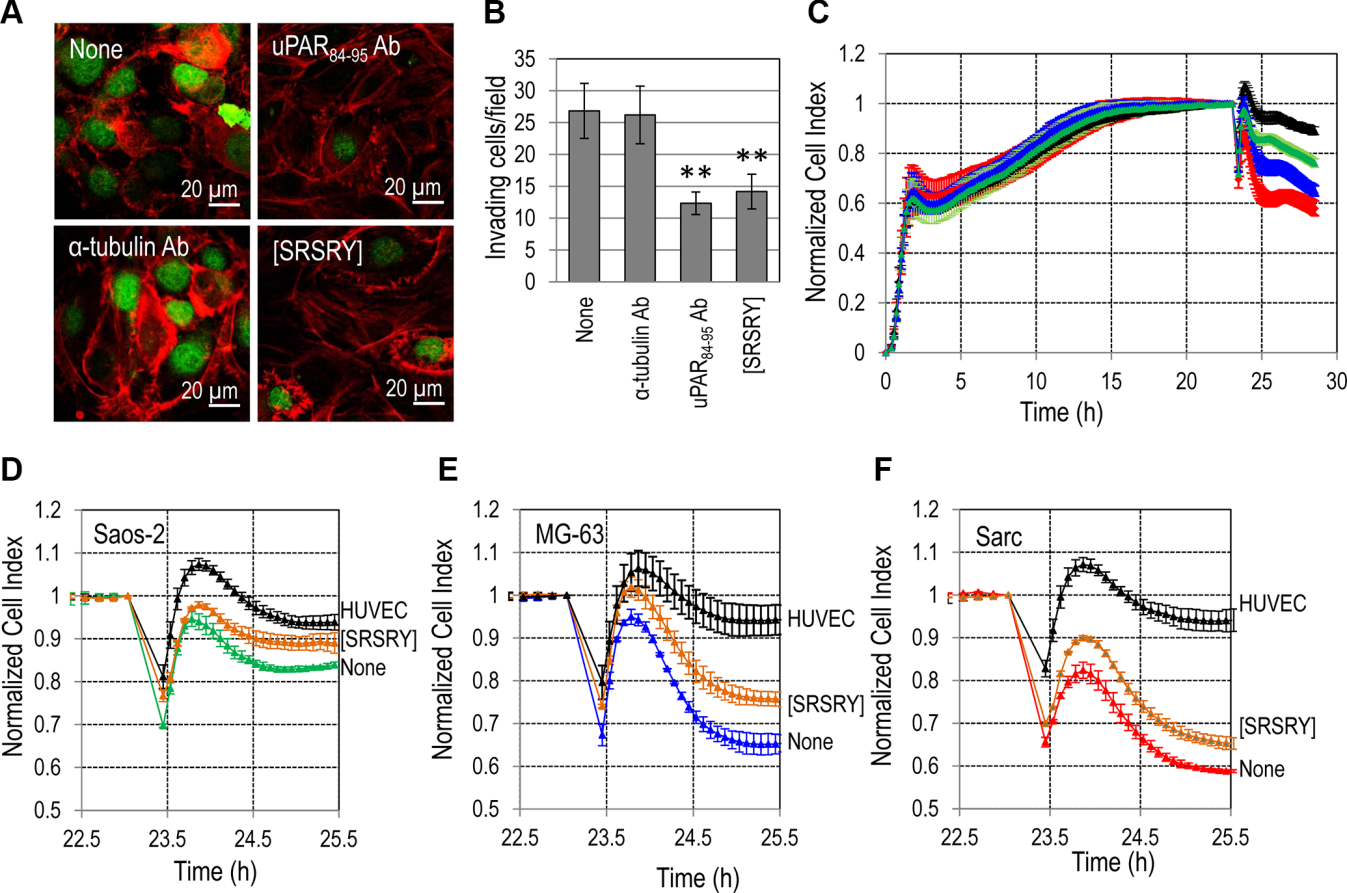
Inhibitory effect of [SRSRY] on trans-endothelial migration of osteosarcoma and chondrosarcoma cells (**A–B**) HUVEC were seeded onto matrigel and allowed to attach and to growth for 24 h prior to seeding GFP-Sarc cells suspended in complete endothelial medium plus/minus diluents (None), 10 nM [SRSRY], anti-uPAR_84-95_ Ab or anti-α-tubulin control Ab for 2 h at 37°C, 5% CO_2_. A. Representative images stained with rhodamine- phalloidin and recorded at a single *plane* confocal to the endothelial monolayer. Scale bar: 10 μm. Original magnifications: 630x. (**B**) GFP-Sarc cells were counted on multiple z-series collected at 0.25 μm intervals using a confocal microscope (Carl Zeiss). The experiments were performed three times. Statistical significance was calculated against None with **p < 0.001. (**C**) HUVEC suspended in growth medium, were seeded in E-16-well plates and allowed to grow for 20-25 h until they form a confluent monolayer (black line), prior to seeding Saos-2 (green line) MG-63 (blue line) or Sarc (red line) cells in growth medium. When HUVECs are challenged with crossing cells, there is a drop in electrical resistance which is monitored in real-time for ~2 h as the cell index changes due to the rupture of endothelial monolayer. (**D–F**) With the same experimental design, Saos-2 (D), MG-63 (E), and Sarc (F) cells plus/minus 10 nM [SRSRY] were allowed to invade endothelial monolayer. In all cases, data represent mean ± SD from a quadruplicate experiment.

The entry of tumor cells into bloodstream is one of the earliest events of the metastatic process. To ascertain if [SRSRY] prevents adhesion onto endothelium and/or trans-endothelial migration of Sarc cells, we performed experiments seeding Green Fluorescent Protein (GFP)-tagged Sarc cells on an endothelial monolayer, labeling co-cultures for F-actin and recording images by a confocal microscope. Analysis of a single plane confocal to the endothelial monolayer revealed numerous Sarc cells interacting with HUVECs, that decreased upon addition of 10 nM [SRSRY] or anti-uPAR_84–95_ but not anti α-tubulin antibodies (Figure 5A). Z-stack analysis of confocal images recorded with 0.25 μm intervals through the entire thickness of the endothelial monolayer, revealed that the majority of Sarc cells are localized underneath the endothelium in the absence of any treatment and that their number was not changed by the addition of the indifferent anti-α-tubulin Ab (26.8 +/− 4 and 26.2 +/− 6 cells/field, respectively). *Vice-versa*, a 55% and 48% reduction of GFP-Sarc cell number was achieved by the addition of anti-uPAR_84–95_ Ab or 10 nM [SRSRY], respectively, (Figure 5B). These data suggest that [SRSRY] prevents both attachment to endothelium and trans-endothelial migration of Sarc cells. To further ascertain if [SRSRY] affects trans-endothelial migration of osteosarcoma and chondrosarcoma cells, the ability of Saos-2, MG-63 and Sarc cells to cross an endothelial monolayer was analyzed using the xCELLigence RTCA technology as described [28]. HUVECs were allowed to grow until they formed a monolayer (~24 h) prior to seeding cells in the presence of 10% FBS plus/minus 10 nM [SRSRY]. At this time, reduction of impedance values, due to invading cells that interrupt monolayer was monitored in real-time for at least 2 h. An about 15%, 30% and 45% reduction of endothelial monolayer integrity was achieved by Saos-2, MG-63 and by Sarc cells, respectively, (Figure 5C). The addition of 10 nM [SRSRY] inhibited the capability of Saos-2 (Figure 5D), MG-63 (Figure 5E) and Sarc (Figure 5F) cells to cross endothelial monolayers by 47%, 38% and 25% respectively. All together, these findings indicate that [SRSRY], at a 10 nM concentration, prevents *in vitro* the extracellular matrix invasion, the formation of a capillary network and the entry into bloodstream.

### The peptide [SRSRY] prevents chondrosarcoma growth, intra-tumoral microvessel density and release of CTCs in the blood of nude mice

**Figure 6:**
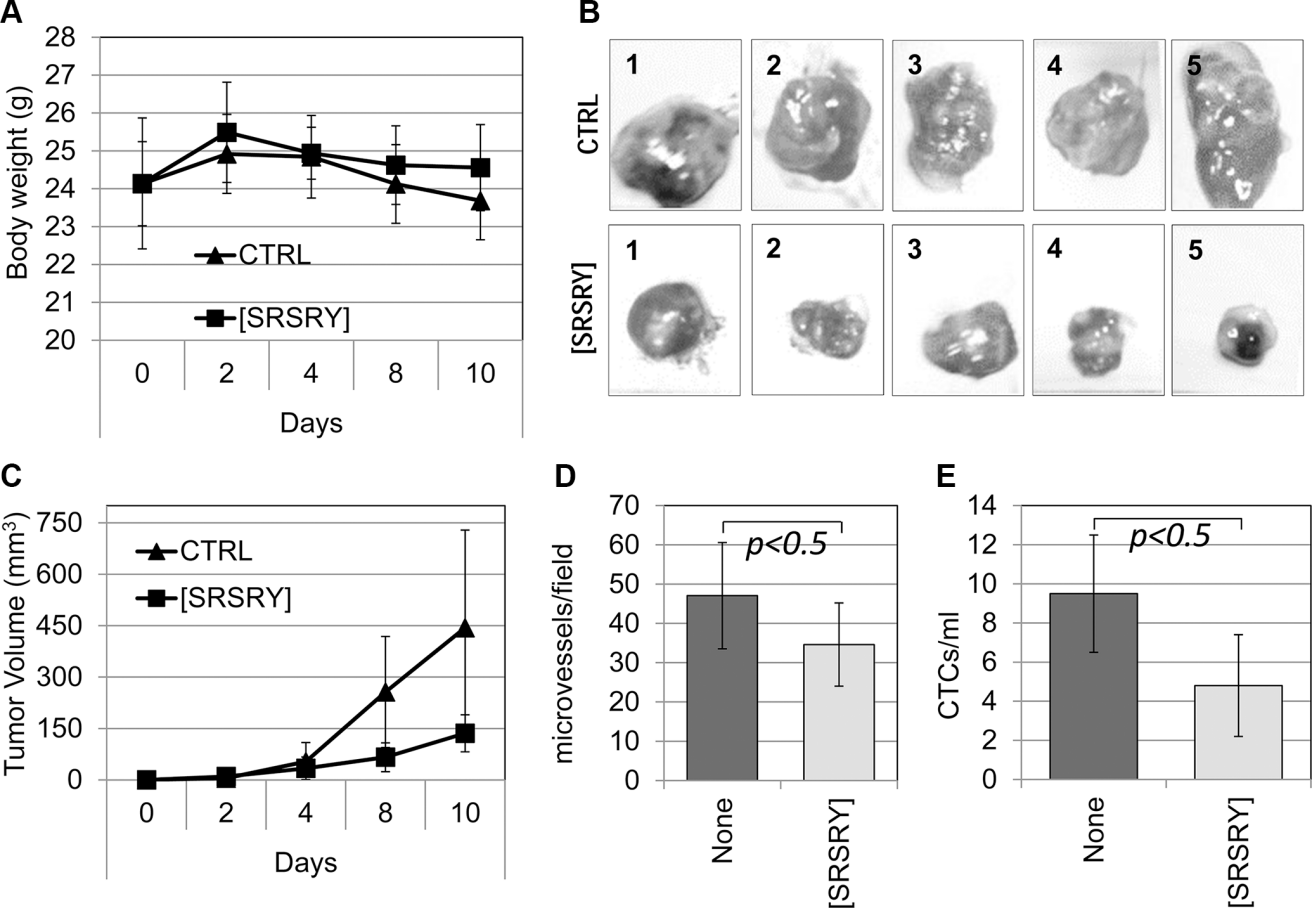
Inhibitory effect of [SRSRY] on the growth, vascularization and invasion of Sarc cells injected in nude mice Ten six-eight week old, Foxn1nu/nu female nude mice of 23 to 25 g, received an injection of Sarc cells into the right flank as a single-cell suspension (1 × 10^6^ cells in 100 μl of sterile PBS, 96% viability). Five animals received i.p. injection of 6 mg/kg peptide [SRSRY] every 24 h and five received injections of vehicle only (CTRL). After 10 days, mice were anesthetized, subjected to retro-orbital blood collection (500 μl/mouse) and then sacrificed by CO_2_ inhalation. (**A**) Animals survived to the treatment schedule without clear changes in body weight. (**B**) After sacrifice of untreated (CTRL) and treated [SRSRY] mice, the image of each excised tumor was acquired. (**C**) Tumor volumes were measured by a caliper every 2 days using the formula [length (mm) × width (mm^2^)/^2^], where the width and the length are the shortest and the longest diameters of each tumor, respectively. (**D**) Microvessel density was assessed by counting vessels on CD-31 immunostained sections in 5 randomly chosen fields per section, in at least two sections per tumor at × 200 magnification. (**E**) Blood samples were collected from mice just before sacrifice and nucleated cells were subjected to DNA extraction and real-time quantitative PCR using primers capable of amplifying ALU sequences. Number of CTCs was calculated by spiking experiments included in the run.

To study the effect of [SRSRY] on tumor growth, ten six-eight week old, Foxn1nu/nu female nude mice (Harlan) received an injection of human Sarc cells into the right flank as a single-cell suspension (1 × 10^6^ cells in 100 μl PBS, 96% viability). Five animals received i.p-administration of 6 mg/Kg [SRSRY] every day for 10 days, and five received injections of vehicle only. Mice survived to the treatment schedule without clear changes in body weight (Figure 6A). Sarc cells readily formed tumors when injected subcutaneously in the flanks of the immuno-compromised mice (Figure 6B). The measurement of tumor volume at various time points showed that the kinetics of tumor formation in vehicle-treated mice were significantly higher than those assessed in [SRSRY]-treated mice (Figure 6C). After 10 days, tumor volumes of vehicle- and [SRSRY]-treated mice were 445+/−285 and 136+/−54 mm^3^, respectively, with *p* < 0.05. Inhibitory effect of [SRSRY] on tumor growth is not due to a reduced proliferation rate, because, *in vitro*, 10 μM peptide did not modify cell growth up to 92 h (Supplementary Figure S1). However, according with the ability of [SRSRY] to prevent *in vitro* formation of a capillary network (Figure 4), we found that microvessel density was reduced in tumors from animals treated with [SRSRY] as compared to those treated with vehicle alone (Figure 6D and Supplementary Figure S2). Circulating Tumor Cells (CTC)s released into the bloodstream from solid tumors, are considered markers of the metastatic process and [SRSRY] prevents trans-endothelial invasion by Sarc cells. Thus, we quantified the CTCs released in the blood samples collected just before the sacrifice of untreated and treated mice. DNA from nucleated cells of murine blood samples was purified and quantitated by Real-Time PCR using primers targeting human Alu-sequences. Number of CTCs was calculated by comparing the obtained amplification curves with others generated in spiking experiments which were included in every run. We found 9,4 +/− 3 CTCs/mL blood samples from 5/5 untreated mice and 4,8 +/− 2 CTCs/mL blood from 4/5 mice treated with [SRSRY] (Figure 6E). All together, these findings indicate that [SRSRY] prevents three key events occurring during the metastatic process of osteosarcoma and chondrosarcoma cells: the extracellular matrix invasion, the formation of a capillary network and the entry into bloodstream.

## DISCUSSION

Beyond the initial acquisition of invasiveness in primary tumors, the next major rate-limiting step in the metastatic cascade is intra-vasation of tumor cells into circulation. Indeed, despite significant progress regarding chemotherapy and improvements in the outcome for patients with localized osteosarcoma or chondrosarcoma, patients who have metastases at diagnosis are not uncommon, and still have poor prognosis [1–2, 29]. This suggests that, at the time of their initial diagnosis, clinically undetectable tumor had already spread to distant sites and that an effective systemic anti-metastatic cancer therapy is needed.

In this study, we present evidence that the cyclization of the SRSRY sequence of uPAR generates a new potent inhibitor of osteosarcoma and chondrosarcoma cell invasion. [SRSRY] prevents three key events occurring during the metastatic process of osteosarcoma and chondrosarcoma cells: the extracellular matrix invasion, the formation of a capillary network and the entry into bloodstream. When chondrosarcoma cells were subcutaneously injected in nude mice, and [SRSRY] was daily administered at 6 mg/Kg, it was apparently well tolerated as weights of mice injected with vehicle or vehicle containing [SRSRY] were comparable. Also, we did not notice the occurrence of adverse side effects that might hamper the therapeutic potential of [SRSRY]. Moreover, tumor size, intra-tumoral microvessel density and circulating tumor cells in blood samples, collected before the sacrifice, were significantly reduced in animals treated with [SRSRY] as compared to animals treated with vehicle only.

The uPAR is a widely recognized master regulator of cell migration and plays an important role in sarcoma cell invasion [7–9]. However, to date, most therapeutic strategies targeting uPAR have not shown robust anti-tumor activity [30]. uPAR participates to a complex signaling network that control cancer progression, providing a basis for the development of new therapies targeting uPAR interactors [13]. A possibility is to interfere with the uPAR/FPR1 interaction. Its uPAR_88–92_ sequence is the minimal region required to induce cell motility and angiogenesis by interacting with the formyl peptide receptor type 1 (FPR1) [17, 21]. Human FPR1, originally identified in neutrophils, monocytes and macrophages, elicits many responses upon ligation of formyl-peptide ligands derived from bacteria and mitochondria, including morphological polarization, locomotion, production of reactive-oxygen species and release of cytokines and proteolytic enzymes [31]. Accumulating evidence demonstrates that FPR1 is also involved in the tumor progression of solid tumors [32–35]. Therefore, FPR1 is a potential therapeutic target for the treatment of malignant human cancer. Agonist binding to FPR1 elicits a signal transduction cascade involving phosphatidylinositol 3-kinase, protein kinase C, AKT and MAPK [35, 36]. Furthermore, it has been shown that inhibition of FPR1-triggered ERK1/2 phosphorylation reduces nuclear translocation of HIF-1α in glioblastoma cells [34–35]. Being GPI-anchored, and therefore lacking transmembrane and intracellular domains, uPAR must cooperate with transmembrane receptors to activate intracellular signalling. Upon binding to FPR, soluble forms of uPAR containing uPAR_84–95_ sequence or the SRSRY peptide promote cell migration and angiogenesis by activating vitronectin receptor with an inside-out type of mechanism which involves PKC and ERK phosphorylation [17, 21]. In the last decade, it has been suggested that unengaged uPAR may exist in a latent inactive form that, upon binding to urokinase, may be subjected to a conformational change shifting the uPAR structure to an active conformation [37–38]. We found that only the active form of uPAR interacts with FPR1 thus inducing cell migration and that the substitution of Ser90 in the uPAR chemotactic sequence with a glutamic acid residue prevents agonist-triggered FPR1 activation and internalization leading to the inhibition of uPAR/vitronectin receptor association and ATF-induced AKT phosphorylation [39]. Thus, it is conceivable to hypothesize that [SRSRY]-inhibitory effect is mediated by FPR1 which, in turn, regulates integrin activity by modulating directly or indirectly multiple signaling pathways.

With the aim to inhibit the functions of uPAR, we previously developed a series of linear peptides that inhibit uPAR-FPR1 interaction, fMLF-induced FPR1 internalization and ERK1/2 phosphorylation, reducing to the basal level directional cell migration [5, 40–41]. However, many of these peptides are unstable to enzymatic digestion, which limits their half-life *in vivo*. In this study, we found that osteosarcoma and chondrosarcoma cells express comparable levels of FPR1 on cell surface and that the peptide [SRSRY] that displays a long-time resistance to enzymatic digestion in serum [25], inhibits, at 10 nM concentration, both migration and invasion of osteosarcoma and chondrosarcoma cell lines exposed to growth medium. The mechanism by which the cyclic peptide [SRSRY] interferes with fMLF binding to FPR1 has been investigated in rat basophilic leukaemia RBL-2H3/ETFR cells expressing high levels of constitutively activated FPR1: [SRSRY] inhibits fMLF- induced, FPR1-mediated cell migration by blocking both internalization and fMLF- and SRSRY-uptake of FPR1 [25]. Now we found that [SRSRY] at 10 nM concentration reduces to the basal level SRSRY-induced cell migration of osteosarcoma and chondrosarcoma cell lines.

The peptide [SRSRY] also reduces the capability of osteosarcoma and chondrosarcoma cells to cross endothelial monolayers, although to a different extent. Extension of blood vessels from preexisting vascular structures and *de novo* formation of vessel networks through the recruitment of bone marrow-derived precursor cells are the essential process for sustained tumor growth and provides the systemic network that stimulates metastasis [3]. Here we report that [SRSRY] prevents *in vitro* tube formation by endothelial cells exposed to conditioned medium of chondrosarcoma cells. According to the finding that Sarc cells express high levels of uPAR on cell surface and release a larger amount of SuPAR [8], these tube-like structures were reduced almost to basal levels by the anti-uPAR_84–95_ polyclonal antibody. However, since chondrosarcoma cells have been reported to secrete VEGF [42] and glioblastoma cells bearing FPR1 produce VEGF in response to fMLF [43], it will be interesting to investigate the possibility that [SRSRY] exerts such effect on VEGF-triggered angiogenesis. This would not be surprising because we have previously reported that inhibitors of uPAR/FPR1 are able to prevent VEGF-driven angiogenesis [27]. Moreover, the decreased vascularization observed in tumors formed by Sarc cells implanted in nude mice treated with [SRSRY], clearly support the inhibitory activity of [SRSRY] on angiogenesis. Indeed, the reduced tumor volumes observed in [SRSRY] treated mice cannot be due to changes in the proliferation rate since, *in vitro*, [SRSRY] did not modified doubling time of Sarc cells up to 10 μM.

In this context, the peptide [SRSRY] which is able to interfere with the ability of osteosarcoma and chondrosarcoma cells to cross extracellular matrix, to promote formation of a capillary network and to entry into bloodstream, could be considered a valid prototype for the development of new anti-neoplastic therapies designed to counteract metastatic dissemination of osteosarcoma and chondrosarcoma cells.

## MATERIALS AND METHODS

### Peptide synthesis and purification

Peptides SRSRY and [SRSRY] were synthesized as previously described [25]. Analytical Reversed phase HPLC indicated > 95% purity and the correct molecular ions were confirmed by liquid chromatography-electrospray ionization-tandem mass spectrometry.

### Cell lines

Human osteosarcoma Saos-2 and MG-63 cell lines (purchased from *ATCC)* and chondrosarcoma Sarc cells, the last derived from a chondrosarcoma primary culture [8], were grown in Dulbecco Modified Eagle Medium (DMEM) supplemented with 10% fetal bovine serum (FBS), 100 IU/mL penicillin and 50 μg/ mL streptomycin. Sarc transfectants, stably expressing Green Fluorescent Protein (GFP), were obtained using pEGFP-N1 vector (Clontech) and polyfectamin transfection reagent (Quiagen). G418-resistant cells expressing the highest levels of GFP were isolated and amplified. Human umbilical vein endothelial cells (HUVEC)s, purchased by Lonza, and employed between the third and the seventh passage, were grown in Eagle Basal Medium (EBM) supplemented with 4% FBS, 0.1% gentamicin, 1 μg/mL hydrocortisone, 10 μg/mL epidermal growth factor and 12 μg/mL bovine brain extract (Cambrex, Bio Science).

### Fluorescence microscopy

Cells, seeded on glass slides (30%-40% confluence), were fixed with 2.5% formaldehyde in PBS for 10 min at 4°C, than incubated 2 h at 23°C with 2 μg/mL R4 anti-uPAR monoclonal antibody or 1:100 anti-FPR1 polyclonal antibody (Ab), the first kindly provided by G. Hoyer-Hansen (Finsen Institute, Copenhagen, Denmark), the last purchased from Santa Cruz Biotechnology. Immunofluorescence was carried out by incubating slides with 1:700 diluted Alexa 488-coniugated F(ab')2 fragment of rabbit anti-mouse IgG or goat anti-rabbit IgG (Molecular Probes) 40 min at 23°C. After nuclear staining with 4–6-diamidino-2-phenylindole dye (DAPI), cells were mounted using 20% (w/v) mowiol, and visualized with the Axiovert 200M inverted fluorescent microscope connected to a video camera (Carl Zeiss).

### Western blotting

Cells detached using 200 mg/L EDTA, 500 mg/L trypsin (Cambrex), were lysed in RIPA buffer (10 mM Tris pH 7.5, 140 mM NaCl, 0.1 % SDS, 1% Triton X-100, 0.5% NP40) containing protease inhibitor mixture. Protein content of cell lysates was measured by a colorimetric assay (BioRad). Twenty and forty μicrograms of proteins from each cell lysate were separated on 10% SDS-PAGE and transferred onto a polyvinylidene fluoride membrane. The membranes were blocked with 5% non-fat dry milk and probed with 1 μg/mL R4 anti-uPAR monoclonal antibody recognizing uPAR D3 domain, 1 μg/mL anti-FPR1 polyclonal antibody (Abcam), or 0.2 μg/mL GAPDH Ab (Santa Cruz Biotechnology). Washed filters were incubated with horseradish peroxidase-conjugated anti-mouse or anti-rabbit antibody and detected by ECL (Amersham- GE Healthcare). Densitometry was performed using the NIH Image 1.62 software (Bethesda, MD). Each experiment was performed three times.

### Cell migration in Boyden chamber

Cell migration in Boyden chambers was carried out as described [17]. Briefly, cell suspension (1 x10^5^ viable cells/mL serum free medium) was seeded in each upper chamber. Lower chambers were filled with DMEM alone, DMEM containing 10 nM SRSRY, 10 nM fMLF, or 10% FBS with/without 10 nM [SRSRY]. A subset of experiments were performed on cells desensitized with 100 nM fMLF for 30 min at 37°C in humidified air with 5% CO_2_ as described [21]. The two compartments were separated by 8 μm pore size polycarbonate filters (Neuroprobe) coated with 2.5 μg/mL vitronectin (Corning). Incubation, was carried out for 4 h at 37°C in humidified air with 5% CO_2_. At the end of the assay, cells on the lower filter surface were fixed with ethanol, stained with haematoxylin and 10 random fields/filter were counted at 200x magnification. Each experiment was performed three times in triplicate.

### Migration kinetic of cells monitored in real time

Kinetic of cell migration was monitored in real time using the xCELLigence Real Time Cell Analysis (RTCA) technology (Acea Bioscience) as described [28]. For these experiments we used CIM-16-well plates which are provided with interdigitated gold microelectrodes on bottom side of a filter membrane interposed between a lower and an upper compartment. The lower chamber was filled with serum-free medium or growth medium with/without 10 nM [SRSRY]. Cells (2 × 10^4^ cells/well) were seeded on filters in serum-free medium. Microelectrodes detect impedance changes which are proportional to the number of migrating cells and are expressed as cell index. Migration was monitored in real-time for 12 h. Each experiment was performed at least twice in quadruplicate.

### Invasion kinetic of cells monitored in real time

This assay was performed using E-16-well plates and the xCELLigence RTCA technology as described [28]. Bottom wells were coated with 20 μg/well matrigel diluted in serum free medium. Matrigel was allowed to polymerize for 1 h at 37°C prior to seeding cells (1 × 10^4^ cells/well) suspended in growth medium plus/minus 10 nM [SRSRY]. Cells that cross matrigel adhere to the bottom of plates causing impedance changes which are proportional to the number of invading cells. Matrigel invasion was monitored in real-time for 18 h. The impedance value of each well was automatically monitored and expressed as a cell index value. The experiments were performed three times in in quadruplicate.

### Cell proliferation

Cell proliferation of Sarc cells was assessed using the xCELLigence technology as described [28]. Briefly, cells (2 × 10^3^/well) were seeded in E-16-well plates in growth medium and left to growth for 92 h in the presence or the absence of 10 μM [SRSRY] or diluents. Microelectrodes placed on the bottom of plates, detect impedance changes which are proportional to the number of adherent cells and are expressed as cell index. Growth medium with/without [SRSRY] was replaced every 24 h. The experiments were performed twice in quadruplicate.

### Tube formation in a non-contact co-culture system

Sarc cells were grown to 80% confluence (1.5 × 10^5^ cells/well) on 24 well plates and kept serum free for 18 h prior to the experiment. Growth factor reduced matrigel (100 μl/well) (Becton Dickinson, cat. 356230) was allowed to polymerize on a polyester membrane in an intercup chamber. Subsequently, the intercup chamber was introduced in the wells filled with DMEM or conditioned medium of Sarc cells. When indicated, 4 μg/ml rabbit anti-uPAR_84–95_ polyclonal antibody, 4 μg/mL α-tubulin polyclonal antibody or 10 nM [SRSRY] were added to well and kept during the assay. HUVEC (2 × 10^4^ cells/sample) were seeded on matrigel at 37°C, 5% CO_2_ for 4 h. To quantify tube formation five random areas/well at 100x magnification were imaged and the number of tubes formed by cord-like structures exceeding 100 μm in length [21], measured using Axiovision 4.4 software (Carl Zeiss), were counted. The experiments were performed three times in duplicate.

### Trans-endothelial migration

To assess the ability of Sarc cells to adhere onto and/or cross endothelium, GFP-tagged Sarc cells were seeded on an endothelial monolayer as previously described [25]. Briefly, sterile round glass coverslips (12 mm in diameter) were coated with 1:8 diluted matrigel (Becton Dickinson). HUVEC (5×10^4^ cells in 200 μL/well) were seeded onto matrigel and allowed to attach and to growth for 24 h at 37°C, 5% CO_2_ prior to seeding GFP-Sarc cells (1 × 10^4^ cells/well) suspended in complete endothelial medium plus/minus diluents, 10 nM [SRSRY], 4 μg/ml anti-uPAR_84–95_ or 4 μg/mL anti-α-tubulin antibodies for 2 h at 37°C, 5% CO_2_. Then, slides were fixed and permeabilized with 2.5% formaldehyde-0.1% Triton X-100 in PBS for 10 min at 4°C, washed in PBS and then incubated with 0.1 μg/mL rhodamine-conjugated phalloidin (Invitrogen) at 23°C for 45 min. Finally, GFP-Sarc cells were identified and counted on multiple z-series collected at 0.25 μm intervals using a confocal microscope (Carl Zeiss). Trans-endothelial migration assays were performed using the xCELLigence RTCA technology as described [28]. Briefly, HUVECs (1×10^4^ cells/well) suspended in growth medium, were seeded in E-16-well plates and allowed to grow for ~24 h until they form a confluent monolayer, prior to seeding osteosarcoma or chondrosarcoma cells (1×10^4^ cells/well) in growth medium plus/minus 10 nM [SRSRY]. When HUVECs are challenged with crossing cells, there is a drop in electrical resistance which is monitored in real-time for 2 h as the cell index changes due to crossing of the endothelial monolayer. The experiment was performed twice in quadruplicate.

### Growth and vascularization of tumors in mice

To evaluate the effect of [SRSRY] on tumor growth and vascularization, Sarc cells were injected, as a single-cell suspension (1 × 10^6^ cells in 100 μl of sterile PBS, 97% viability), subcutaneously in the flanks of ten six-eight week old, Foxn1nu/nu female nude mice (Harlan). Animals were randomized into two groups of five with the treatment group receiving 6 mg/kg [SRSRY] by intra-peritoneal injection every 24 h and the control group receiving an equivalent injected volume of vehicle (PBS) only. Time-dependent average weight was monitored every two days. The length and the width of the tumors were measured at different time points with the help of a calliper and the volume was calculated using the formula: ½ × (width)^2^ × length (mm). After 10 days, blood samples (at least 500 μL/mouse) from the retroorbital venous plexus of mice anesthetized with 1% isoflurane were collected using a heparinized capillary tube and processed for determination of the Circulating Tumor Cells (CTC)s. Then, animals were sacrificed and the excised tumors were fixed in buffered formalin and processed for paraffin sectioning. Tumor vascularization was assessed by counting vascular channels harbouring red blood cells on CD31 immuno-stained sections in 5 randomly chosen fields per section, in at least two sections per tumor at x 200 as described [28].

### Isolation and enumeration of CTCs

To quantify CTCs, DNA from nucleated cells of murine blood samples (500 μl/mouse) was purified using the QIAamp DNA Mini Kit (Qiagen), according to the manufacturer's protocol. Quantitative RT-PCR (7900 HT Fast Real-Time PCR System, Applied Biosystems) was performed using 18 ng DNA and the SYBR Select Master Mix (Applied Biosystems). Primers targeting human Alu-sequences [FW 5′- CACCTGTAATCCCAGCACTTT-3′/RW 5′-CCCAGGCTGGAGTGCAGT-3′] were employed to a final concentration of 0.5 μM. The number of CTCs was calculated by comparing the obtained CT with a standard amplification curve generated in spiking experiments (1 to 50 cells were collected by pipetting under microscopic control) which were included in every run. DNA from murine blood was included as a negative control.

### Statistical analysis

The data were analysed for significance using Student's *t*-test. Differences were considered statistically significant at a level of *p <* 0.05.

### Ethics statement

The research work with mouse model has been approved by Institutional Ethical Committee of Istituto Nazionale Tumori “Fondazione G. Pascale”-IRCCS, Naples, Italy (protocol n. 09, December 20th, 2010).

## SUPPLEMENTARY MATERIALS



## Abbreviations

uPAR: urokinase receptor
ECM: extracellular matrix
fMLF: N-formyl-Met-Leu-Phe
FPR: formyl-peptide receptor
FBS: fetal bovine serum
PBS: phosphate-buffered saline
GFP: green fluorescent protein
HUVEC: human umbilical vein endothelial cell
CTC: circulating tumor cells
