# Supplementary material for: The urokinase receptor-derived cyclic peptide [SRSRY] suppresses neovascularization and intravasation of osteosarcoma and chondrosarcoma cells
Source: Oncotarget. 2016 Jun 13;7(34):54474–87. doi: 10.18632/oncotarget.9976 (PMC5342356; doi:10.18632/oncotarget.9976)
Supplement: Supplementary file 1 [file oncotarget-07-54474-s001.pdf]

The urokinase receptor-derived cyclic peptide [SRSRY] suppresses neovascularization and intravasation of osteosarcoma and chondrosarcoma cells

Supplementary Materials

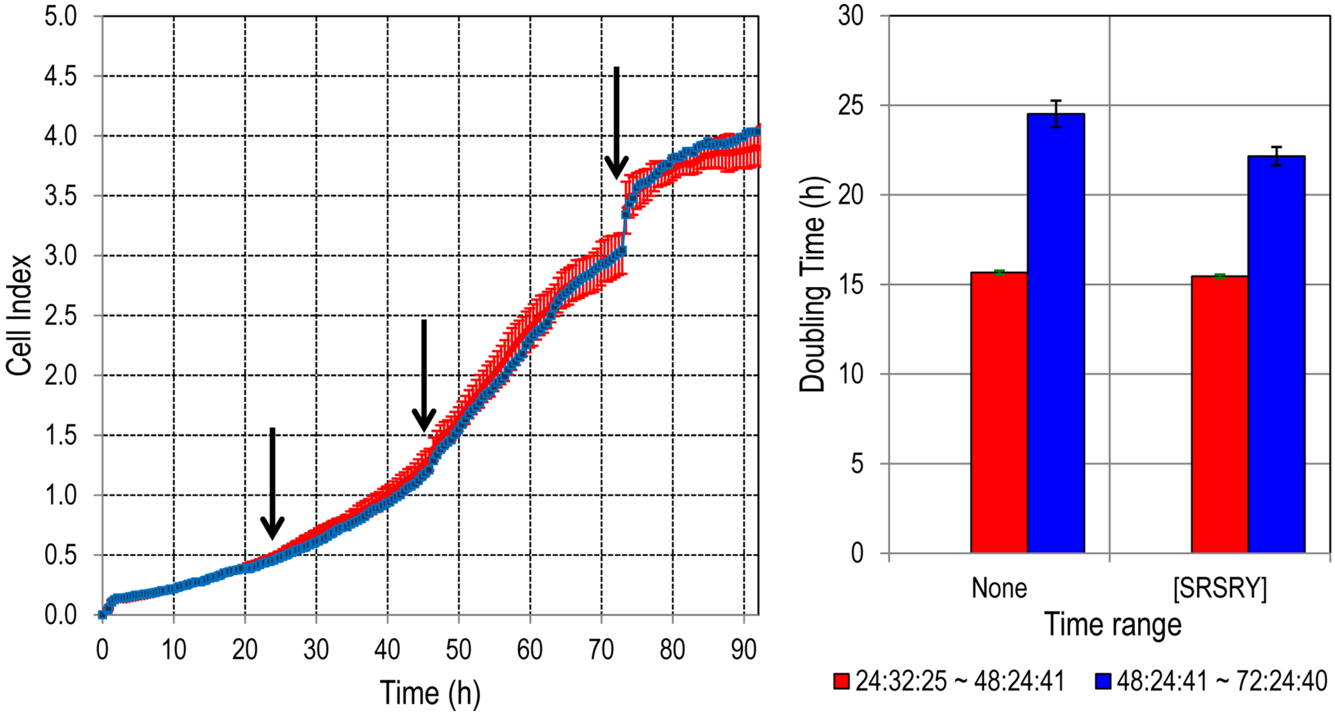

Supplementary Figure S1: Cell proliferation of Sarc cells exposed to 10 µM [SRSRY]. Cell proliferation of Sarc cells exposed to 10 µM [SRSRY], assessed by monitoring impedance by RTCA xCELLigence system. Growth medium with (red) or without (blue) 10 µM [SRSRY] was replaced every 24 h (arrows). Data represent mean ± SD from a quadruplicate experiment.

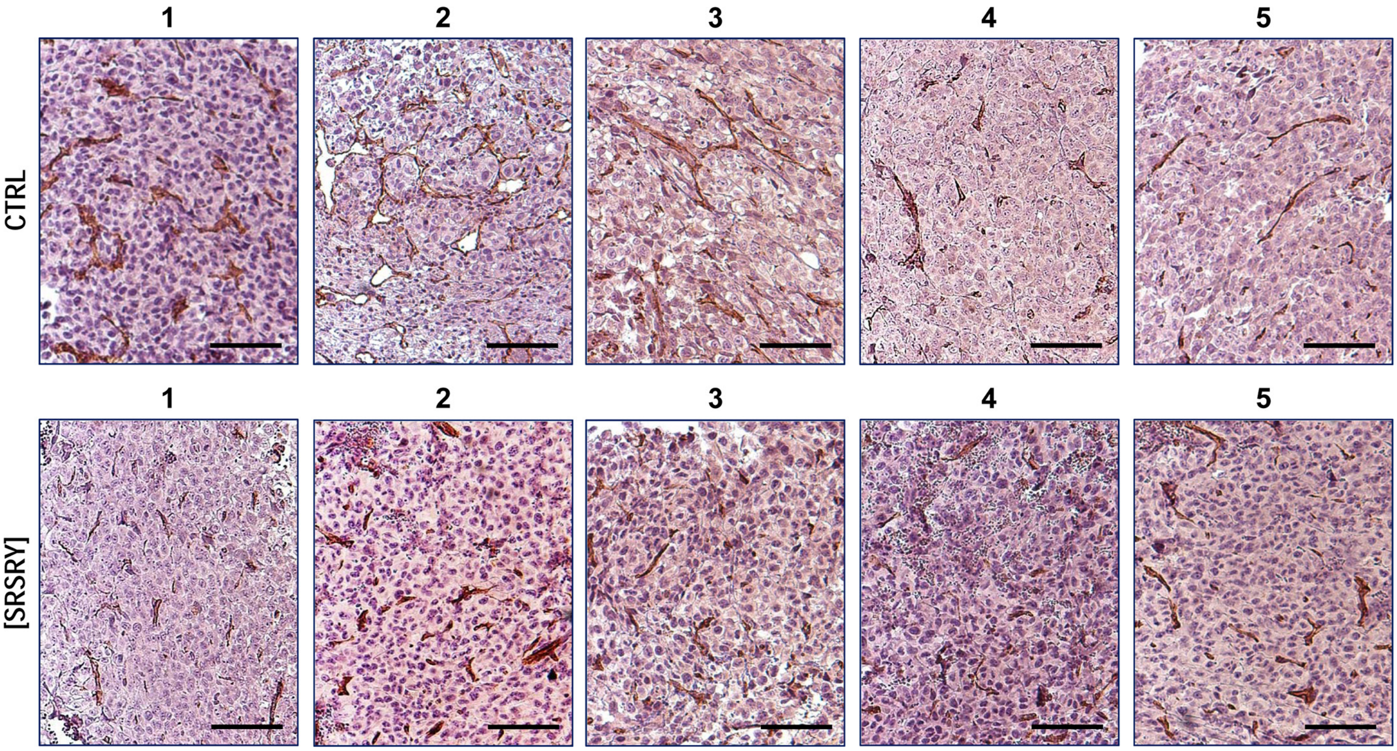

Supplementary Figure S2: Tumor vascularization of primary tumor nodules. Vessels were counted on CD-31 immunostained sections in 5 randomly chosen fields per section, in at least two sections per tumor at x 200 magnification. Scale bar: 50 µm. Original magnification: 200x.
